# Short-hairpin RNA-induced suppression of adenine nucleotide translocase-2 in breast cancer cells restores their susceptibility to TRAIL-induced apoptosis by activating JNK and modulating TRAIL receptor expression

**DOI:** 10.1186/1476-4598-9-262

**Published:** 2010-09-28

**Authors:** Ji-Young Jang, Yoon-Kyung Jeon, Yun Choi, Chul-Woo Kim

**Affiliations:** 1 Department of Pathology, Tumor Immunity Medical Research Center, Cancer Research Institute, Seoul National University College of Medicine, 28 Yongon-dong, Jongno-gu, Seoul 110-799, South Korea

## Abstract

**Background:**

Tumor necrosis factor (TNF)-related apoptosis-inducing ligand (TRAIL; apo2 ligand) induces apoptosis in cancer cells but has little effect on normal cells. However, many cancer cell types are resistant to TRAIL-induced apoptosis, limiting the clinical utility of TRAIL as an anti-cancer agent. We previously reported that the suppression of adenine nucleotide translocase-2 (ANT2) by short-hairpin RNA (shRNA) induces apoptosis of breast cancer cells, which frequently express high levels of ANT2. In the present study, we examined the effect of RNA shRNA-induced suppression of ANT2 on the resistance of breast cancer cells to TRAIL-induced apoptosis *in vitro *and *in vivo*.

**Results:**

ANT2 shRNA treatment sensitized MCF7, T47 D, and BT474 cells to TRAIL-induced apoptosis by up-regulating the expression of TRAIL death receptors 4 and 5 (DR4 and DR5) and down-regulating the TRAIL decoy receptor 2 (DcR2). In MCF7 cells, ANT2 knockdown activated the stress kinase c-Jun N-terminal kinase (JNK), subsequently stabilizing and increasing the transcriptional activity of p53 by phosphorylating it at Thr81; it also enhanced the expression and activity of DNA methyltransferase 1 (DNMT1). ANT2 shRNA-induced overexpression of DR4/DR5 and TRAIL sensitization were blocked by a p53 inhibitor, suggesting that p53 activation plays an important role in the transcriptional up-regulation of DR4/DR5. However, ANT2 knockdown also up-regulated DR4/DR5 in the p53-mutant cell lines BT474 and T47 D. In MCF7 cells, ANT2 shRNA treatment led to DcR2 promoter methylation and concomitant down-regulation of DcR2 expression, consistent with the observed activation of DNMT1. Treatment of the cells with a demethylating agent or JNK inhibitor prevented the ANT2 shRNA-induced down-regulation of DcR2 and activation of both p53 and DNMT1. In *in vivo *experiments using nude mice, ANT2 shRNA caused TRAIL-resistant MCF7 xenografts to undergo TRAIL-induced cell death, up-regulated DR4/DR5, and down-regulated DcR2. Co-treatment with ANT2 shRNA and TRAIL efficiently suppressed tumor growth in these mice.

**Conclusions:**

ANT2 suppression by shRNA might be exploited to overcome TRAIL-resistance in cancer.

## Background

Tumor necrosis factor (TNF)-related apoptosis-inducing ligand (TRAIL; also known as apo2 ligand) is a member of the TNF subfamily. TRAIL induces apoptosis by recognizing and binding to its cognate receptors on cell surfaces. These receptors are known as death receptor 4 (DR4; TRAIL receptor 1; TRAILR1) and death receptor 5 (DR5; TRAIL receptor 2; TRAILR2). Binding initiates conformational changes in the receptors and recruits an adaptor molecule (Fas-associated death domain) and initiator caspases (caspase-8 and -10) to form a death-inducing signaling complex. This process activates caspase-8 and -10, which can then directly activate effector caspases (caspase-3, -6, and -7) to cause apoptosis. Alternatively, activated caspase-8 and -10 can cleave Bid protein to engage the intrinsic apoptotic pathway through mitochondria. However, TRAIL can also bind to decoy receptors 1 (DcR1; TRAILR3) and 2 (DcR2; TRAILR4) on cell surfaces; these decoy receptors function as dominant-negative forms and protect cells from apoptosis by competing with the death receptors for TRAIL interaction [1,2].

Because TRAIL can induce apoptosis in cancer cells but has little effect on normal cells, it is considered a promising anticancer agent [1,2]. TRAIL-based therapies, including recombinant human TRAIL and DR4/DR5-specific agonistic monoclonal antibodies, are currently undergoing phase I and II clinical trials [3]. However, the anticancer applications of TRAIL are unfortunately limited by the fact that cancer cells are often resistant to TRAIL-induced cell death [4,5]. This resistance is conferred by a number of molecular changes, such as the reduced expression of death receptors; the elevated expression of anti-apoptotic molecules, including decoy receptors, FLICE-like inhibitory protein (FLIP), X-linked inhibitors of apoptosis proteins (XIAPs), anti-apoptotic Bcl-2-family proteins; and NF-κB activation [6,7]. Many efforts have been made to identify strategies to overcome those TRAIL resistance mechanisms. In fact, combination therapies using recombinant TRAIL or agonistic anti-TRAIL receptor monoclonal antibodies together with other anti-cancer agents have shown improved efficacy for cancer treatment *in vitro *and *in vivo *through modulation of TRAIL-resistant mechanisms [7]. Some of the TRAIL-sensitizing agents investigated have included histone deacetylase inhibitors [8], cyclin-dependent kinase inhibitors [9], proteasome inhibitors [10], Myc oncoproteins, and Raf kinase inhibitor [11]. Several phytochemicals, such as luteolin, also appear to be effective at overcoming TRAIL-resistance *via *degradation of XIAP [12], and 3,3-diindolymethane down-regulates FLIP [13]. In addition, proteins of the Bcl-2 family, which are key regulators of apoptosis through the intrinsic mitochondrial pathway, are often deregulated in cancers and can be manipulated to achieve TRAIL sensitization [14,15].

Adenine nucleotide translocase, a protein located in the inner mitochondrial membrane, catalyzes the exchange of mitochondrial ATP with cytosolic ADP and participates in the formation of the mitochondrial permeability transition pore complex that interacts with Bcl-2-family proteins. Adenine nucleotide translocase-2 (ANT2), one of the four adenine nucleotide translocase isoforms expressed in humans, is expressed at high levels in undifferentiated cells and tissues with high proliferating and regenerating capacity, including lymphocytes, kidney, and liver [14-16]. Recently, ANT2 was found to be over-expressed in hormone-dependent cancers, including breast and ovarian cancers [17]. The induction of ANT2 in cancer cells is associated with glycolytic metabolism, suggesting a role for ANT2 in carcinogenesis [18-24].

We previously observed that the suppression of ANT2 by interference (RNAi) using vector-based short-hairpin RNA (shRNA) disrupted the mitochondrial membrane potential and induced apoptosis in human breast cancer cells and that it inhibited tumor growth in an *in vivo *xenograft model [16]. Moreover, suppression of ANT2 by shRNA modified the balance of Bcl-2 family proteins in mitochondrial membranes to favor a pro-apoptotic status [16]. These findings have led us to investigate the therapeutic potential of ANT2 RNAi in combination with TRAIL.

In the present study, we investigated the effect of ANT2 shRNA treatment on TRAIL-resistant breast cancer cells. We found that it sensitized the cells to TRAIL, greatly increasing their apoptotic response to TRAIL *in vitro *and *in vivo *by up-regulating DR4 and DR5 expression and down-regulating DcR2 expression. These effects were mediated by activation of c-Jun N-terminal kinase (JNK), subsequent p53 activation, and DcR2 gene methylation. Our results suggest that ANT2 shRNA might be useful as a new TRAIL-sensitizing agent in human breast cancer.

## Methods

### Cell lines and culture

The human breast cancer cell lines MCF7, MDA-MB-231, T47 D, and BT474 were purchased from the American Type Culture Collection (Manassas, VA, USA) and cultured in Dulbecco's Modified Eagle Medium supplemented with 10% fetal bovine serum, 100 units/ml penicillin, and 100 μg/ml streptomycin (Gibco, Grand Island, NY, USA) in a humidified 5% CO_2_/95% air atmosphere at 37°C.

### Antibodies and reagents

Antibodies against human DR4 (TRAILR1), DR5 (TRAILR2), DcR1 (TRAILR3), and DcR2 (TRAILR4) were purchased from R&D Systems (Minneapolis, MN, USA). Antibodies against p53 and Thr81-phosphorylated p53 were from Santa Cruz Biotechnology (Heidelberg, Germany). Antibodies against JNK, phospho-JNK, and β-actin were from Cell Signaling Technology (Beverly, MA, USA). Antibodies against ANT2, poly(ADP-ribose) polymerase (PARP), Bid, cleaved caspase-8, cleaved caspase-9, cleaved caspase-7, cytochrome c, DNA methyltransferase (DNMT1), and cyclooxygenase (COX)-IV were from Abcam Inc (Cambridge, MA, USA). Recombinant human TRAIL was purchased from Peprotech Asia (Rocky Hills, NJ, USA) and the methylation inhibitor 5-aza-2'-deoxycytidine (5-aza-dC) was from Sigma (St Louis, MO, USA). The p53 inhibitor pifithrin-α was purchased from Biovision (Zürich, Switzerland), and the JNK inhibitor SP600125 was from Calbiochem-Novabiochem Corp (San Diego, CA, USA).

### Construction and transfection of ANT2 shRNA expression vectors

The ANT2 shRNA expression vector used to achieve specific down-regulation of ANT2 was described previously [16]. In brief, three kinds of ANT2 small-interfering RNA (siRNA; namely, ANT2 siRNA-1, -2, and -3) designed to be complementary to exon 2 or exon 4 of ANT2 (GenBank accession number NM001152) were synthesized, and DNA vectors expressing the shRNA forms of the siRNAs were generated using pSilencer 3.1-H1 puro plasmids with a TTCAAGAGA linker sequence that forms looped structures (Ambion, Austin, TX, USA). The vector expressing ANT siRNA-1 oligonucleotides (5'-GCAGAUCACUGCAGAUAAGTT-3') was used predominantly throughout this study. A scrambled siRNA (Ambion) with no significant homology to human gene sequences was used as a control to detect nonspecific effects.

For transfection, cells were plated on six-well plates (2 × 10^5 ^cells/well) or 100-mm dishes (2 × 10^6 ^cells/dish) and allowed to adhere for 24 h. Lipofectamine 2000 (Invitrogen Corp., Carlsbad, CA) was used to transfect cells with the pSilencer 3.1-H1 puro scrambled siRNA vector (hearafter, scrambled shRNA) or pSilencer 3.1-H1 puro ANT2 siRNA vector (hearafter, ANT2 shRNA). After 6 h, the culture medium was replaced with fresh complement medium, and the cells were harvested at 24-48 h after transfection. The pcDNA3.1 and pcDNA3.1-ANT2 expression vectors were transfected using a similar method.

### Immunoblotting

Cells were lysed with lysis buffer [5 mM ethylenediamine tetra acetic acid (EDTA), 300 mM NaCl, 0.1% Nonidet P-40, 0.5 mM NaF, 0.5 mM Na_3_VO_4_, 0.5 mM phenylmethylsulfonyl fluoride. and 10 μg/ml each aprotinin, pepstatin, and leupeptin (Sigma)]. After centrifugation at 15,000 × *g *for 30 min, the supernatant fractions were analyzed for protein concentration using Bradford reagent (Bio-Rad Laboratories, Inc., Hercules, CA, USA). Aliquots containing 50-μg total protein were subjected to electrophoresis in 10% SDS-PAGE gels, transferred to polyvinylidene difluoride membranes (Millipore, Bedford, MA, USA), and then incubated with the appropriate antibodies. Immunoblots were visualized using an enhanced chemiluminescence detection system (Amersham Pharmacia Biotech, Uppsala, Sweden).

### Cell viability assay

Cell viability was measured using a CCK8 Cell Counting Kit (Dojindo Molecular Technologies, Kumamoto, Japan). Results of cell viability assays are presented as the mean values of three replicate experiments performed in triplicate.

### Apoptosis assay [annexin V and propidium iodide (PI) staining]

MCF7 cells at approximately 2 × 10^5 ^cells per ml were transfected with ANT2 shRNA or scrambled shRNA for the indicated lengths of time. The transfected cells were harvested, washed twice with phosphate-buffered saline (PBS), then incubated for 15 min at room temperature with a solution of fluorescence isothiocyanate (FITC)-conjugated annexin V (2.5 μg/ml) and PI (5 μg/ml) (BD Pharmingen, San Diego, CA, USA), and analyzed for apoptosis using flow cytometry (Epics XL; Coulter, Marseille, France).

### Reporter gene assay

A p53-luciferase-reporter construct containing many p53 binding motifs was co-transfected with scrambled or ANT2 shRNA into MCF7 cells using Lipofectamine 2000 (Invitrogen Corp.) and cultured for indicated lengths of time. After the culture medium was removed, the cells were rinsed gently with PBS, and 200 μl of lysis buffer was added to each well. The cells were incubated at 4°C for 15-20 min and then subjected to one freeze-thaw cycle to ensure complete lysis. The cell lysates were transferred to pre-labeled microcentrifuge tubes and centrifuged at 12,000 × *g *for 4 min at 4°C. The supernatant fractions containing the cell extracts were recovered and assayed for luciferase activities using a single-sample luminometer (FB12 luminometer; Berthold Detection Systems, Pforzheim, Germany).

### Methylation-specific PCR

Genomic DNA was obtained and purified using QIAamp DNA mini Kits (Qiagen, Hilden, Germany). The unmethylated cytosines in aliquots containing 500 ng DNA were converted to uracil using MethylCode Bisulfite Conversion Kits (Invitrogen Corp.). We modified the manufacturer's instructions by extending the desulfonation step to 30 min to ensure complete conversion of the genomic DNA and by pre-warming the elution buffer to 50°C to increase the DNA yield.

Methylation-specific PCR for DcR2 was performed using the primer pair 5'-TTGGGGATAAAGTGTTTTGATT-3' (sense) and 5'-AAACCAACAACAAAACCACA-3' (antisense) for methylated DNA and the pair 5'-GGGATAAAGCGTTTCGATC-3' (sense) and 5'-CGACAACAAAACCGCG-3' (antisense) for methylated DNA. PCR mixtures contained 1× PCR buffer, 2 mM MgCl_2_, 200 mM dNTPs, 200 nM of each primer, 0.05 U/ml Taq polymerase, and 6 ng/ml converted DNA as template. PCR products were resolved in 2% agarose gels in 1× Tris/borate/EDTA buffer and stained with ethidium bromide.

### DNMT1 activity assay

Nuclear extracts from MCF7 cells transfected for 24 h with ANT2 or scrambled shRNA (control) were evaluated for DNMT1 activity using an EpiQuik Dnmt1 Assay Kit (Epigentek Inc., Brooklyn, NY, USA).

### Animal experiments

To evaluate the anti-tumor effects of ANT2 shRNA and TRAIL *in vivo*, we established tumor models in 6-8-week-old BALB/c nude mice by subcutaneously injecting 5 × 10^6 ^MCF7 cells into their right flanks. Three weeks after tumor inoculation, the mice began receiving intraperitoneal injections of PBS or TRAIL (10 mg/kg) with or without intratumoral injections of scrambled shRNA or ANT2 shRNA vector (100 μg; supplemented 200 μl Lipofectamine 2000) thrice daily for 3 days. Tumor dimensions were measured weekly using a caliper, and tumor volumes were calculated using the formula *m*_1_^2 ^× *m*_2 _× 0.5236, where *m*_1 _and *m*_2 _represent the shortest and longest tumor axes, respectively. At three weeks after tumor challenge, the tumors were less than 300 mm^3 ^in volume. The mice were euthanized when tumors reached 2 cm in diameter or became ulcerated; at 45 days after tumor challenge, the tumor masses were so large in the control and TRAIL groups that the mice were euthanized.

### Statistical analysis

Data were analyzed using the Student's *t *test. Differences are considered statistically significant at *P *< 0.05.

## Results

### ANT2 suppression by shRNA sensitizes breast cancer cells to TRAIL

**Figure 1:**
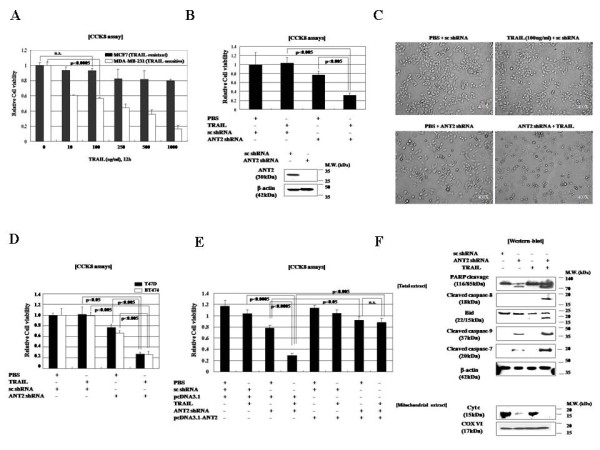
**TRAIL cytotoxicity and expression of TRAIL receptors in the TRAIL-resistant breast cancer cell line MCF7 and the TRAIL-sensitive breast cancer cell line MDA-MB-231**. (A) Cell viability assays were performed 12 h after treatment of MCF7 and MDA-MB-231 cells with recombinant human TRAIL. (B) MCF7 and MDA-MB-231 cells were transfected with 1 μg of scrambled or ANT2 shRNA. After 24 h, they were treated with recombinant human TRAIL (100 ng/ml). Cell viability assays were performed 12 h after TRAIL treatment. (C) Morphologic analysis of TRAIL-resistant MCF7 cells transfected with scrambled or ANT2 shRNA. After 24 h, the cells were treated with recombinant human TRAIL (100 ng/ml) for 12 h and then examined under a phase contrast microscope. (D) The TRAIL-resistant cell lines T47 D and BT474 were treated with shRNA and TRAIL and examined for cell viability as in (A). (E) MCF7 cells were transfected with scrambled or ANT2 shRNA. After 24 h, they were transfected with pcDNA3.1 or pcDNA-ANT2. After another 24 h, they were treated with recombinant human TRAIL (100 ng/ml) for 12 h and assayed for cell viability as in (B). (F) Immunoblot analysis of effect of ANT2 shRNA on TRAIL sensitivity. MCF7 cells were transfected with scrambled or ANT2 shRNA, treated with TRAIL for 12 h as in (A). Cell extracts were prepared, subjected to SDS-PAGE, and immunoblotted with antibodies as indicated.

We first examined the effect of TRAIL on the breast cancer cell lines MCF7 and MDA-MB-231. In CCK8 cell viability assays, MDA-MB-231 cells, but not MCF7 cells, underwent cell death in response to TRAIL (Figure 1A); the MCF7 cells were strongly resistant to TRAIL (Figure 1B). We previously reported that the specific knockdown of ANT2 by RNAi using a DNA vector expressing ANT2 shRNA could induce apoptosis of breast cancer cells [16]. Therefore, to evaluate whether ANT2 suppression increases the TRAIL sensitivity of breast cancer cells, we treated the TRAIL-resistant MCF7 cells with recombinant human TRAIL (100 ng/ml) after transfecting then with ANT2 shRNA. As shown in Figure 1B, transfection of ANT2 shRNA had a slight cytotoxic effect, decreasing the viability of the MCF7 cells by approximately 20%, but treatment of ANT2 shRNA-transfected MCF7 cells with TRAIL caused a much greater decrease in viability (about 70%) than TRAIL or shRNA alone.

Morphologic evaluation of the cells for apoptosis using annexin V/PI staining and flow cytometry confirmed that co-treatment with ANT2 shRNA and TRAIL caused morphologic changes suggestive of apoptosis (Figure 1C) and increased the percentage of annexin V or PI-positive cells (**data not shown**). The TRAIL-resistant breast cancer cell lines T47 D and BT474 also exhibited increased TRAIL sensitivity after transfection with ANT2 shRNA (Figure 1D).

All RNAi experiments included an evaluation of the effects of ANT2 and scrambled shRNA on ANT2 expression by RT-PCR (data not shown) and immunoblotting (Figure 1B). Treatment with the scrambled shRNA control had no influence on TRAIL-induced apoptosis in MCF7 cells, confirming the specific down-regulation of ANT2 by ANT2 shRNA. Other shRNA species directed to different regions of the ANT2 mRNA demonstrated similar TRAIL-sensitizing effects (data not shown). To exclude the possibility of off-target effects of ANT2 shRNA, we used a pcDNA vector to restore ANT2 expression in MCF7 cells transfected with ANT2 shRNA. As shown in Figure 1E, restoration of ANT2 expression reversed the TRAIL-sensitizing effect of shRNA-induced ANT2 knockdown. These findings confirmed that the observed ANT2 shRNA-induced sensitization of breast cancer cells to TRAIL was caused by specific suppression of ANT2 rather than by unforeseen effects of ANT2 shRNA on other genes.

To confirm that TRAIL-induced cell death in ANT2-suppressed MCF7 cells occurs though an apoptotic pathway, we evaluated the effects of ANT2 knockdown and TRAIL on several molecules involved in apoptosis. In immunoblot experiments, co-treatment with TRAIL and ANT2 shRNA elicited caspase-8 and -9 activation, cytochrome c release from mitochondria, and cleavage of PARP (Figure 1F), consistent with induction of apoptosis. Although MCF7 cells are deficient in caspase-3, they remain susceptible to cell death induced by several stimuli including TNF, staurosporine, and various DNA-damaging agents [17]. Consistent with a report that other executive caspases, such as caspase-6 and -7, are able to substitute for caspase 3 during apoptosis, co-treatment with TRAIL and ANT2 shRNA elicited caspase 7 activation in MCF7 cells (Figure 1F). These findings suggest that TRAIL decreases the viability of ANT2 shRNA-treated MCF7 breast cancer cells by inducing apoptosis through a mechanism mediated by caspase-7, -8, and -9 and involving the intrinsic mitochondrial apoptosis pathway.

### ANT2 knockdown up-regulates death receptors and down-regulates decoy receptors, thereby increasing the TRAIL sensitivity of TRAIL-resistant breast cancer cells

**Figure 2:**
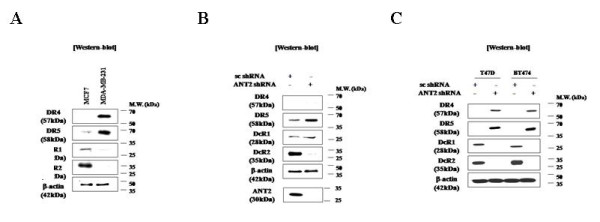
**Effect of ANT2 shRNA on expression of TRAIL receptors in breast cancer cell lines**. TRAIL receptor expression in MCF7 and MDA-MB-231 cells (A), MCF7 cells transfected with scrambled or ANT2 shRNA for 24 h (B), and T47 D and BT474 cells transfected with scrambled or ANT2 shRNA for 24 h (C) was analyzed by SDS-PAGE followed by immunoblotting of cell extracts with antibodies against DR4, DR5, DcR1, DcR2, and β-actin (control) as indicated.

Because TRAIL-resistance in certain cancer cell lines is attributable to attenuated expression of the TRAIL death receptors DR4 and DR5 or over-expression of the decoy receptors DcR1 and DcR2, we examined the expression of these receptors in various breast cancer cell lines. TRAIL-resistant MCF7 cells were found to express very low levels of pro-apoptotic DR4 and DR5 and a very high level of anti-apoptotic DcR2, whereas TRAIL-sensitive MDA-MB-231 cells were found to express high levels of DR4 and DR5 and a very low level of DcR2 (Figure 2A). These data are consistent with those previously reported [15].

These observations led us to assess the effects of ANT2 shRNA transfection on the expression of TRAIL receptors in MCF7 cells. As shown Figure 2B and 2C, ANT2 knockdown dramatically increased DR4 and DR5 expression and decreased DcR1 or DcR2 expression in MCF7 cells, as well as in the TRAIL-resistant cell lines T47 D and BT474. These data suggested that ANT2 shRNA sensitizes MCF7, T47 D, and BT474 cells to TRAIL by shifting the expression of TRAIL death and decoy receptors toward a pro-apoptotic balance.

### ANT2 knockdown up-regulates DR4 and DR5 expression *via *activation of p53

**Figure 3:**
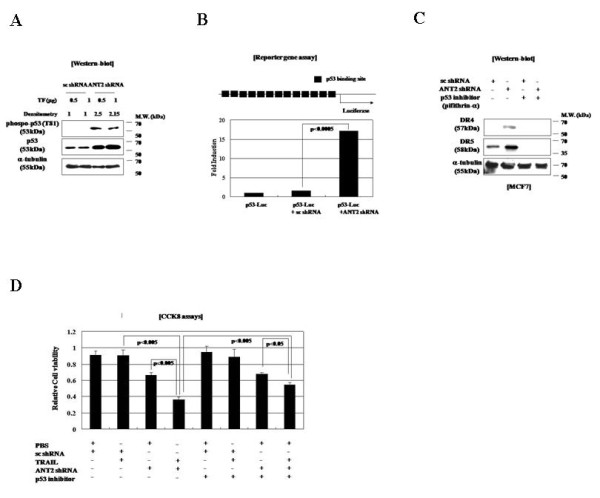
**Up-regulation of DR4 and DR5 expression by ANT2 shRNA-induced activation of p53 in MCF7 cells**. (A) Immunoblot analysis of p53 expression and phosphorylation. MCF7 cells were transfected with scrambled shRNA or ANT2 shRNA. After 24 h, cells extracts were prepared, subjected to SDS-PAGE, and immunoblotted with antibodies against phospho-p53, p53, α-tubulin, ANT2, and β-actin (control). Band intensities were calculated using Image Gauge v4.0 (Fujifilm) (B) Reporter gene analysis of p53 transcriptional activity. MCF7 cells were transfected with a p53-luciferase-reporter construct and scrambled or ANT2 shRNA. After 18 h, cell lysates were prepared and analyzed for luciferase activities using a luminometer. (C) Immunoblot analysis of p53-mediated up-regulation of DR4/DR5. Cells were pre-treated with the p53 inhibitor pifithrin-α for 2 h and then transfected with scrambled or ANT2 shRNA. After 24 h, cell lysates were prepared, subjected to SDS-PAGE, and immunoblotted with antibodies against DR4, DR5, and α-tubulin. (D) Cells were treated with pifithrin-α and scrambled or ANT2 shRNA as in (C) and then analyzed for cell viability using a CCK8 assay kit.

Because the tumor suppressor protein p53 plays an important role in regulating the expression of DR4 and DR5 at the transcriptional level [25], we examined the dependence of ANT2 knockdown-induced up-regulation of DR4 and DR5 on p53 in MCF7 cells. Although ANT2 knockdown did not affect the amount of p53 mRNA in the cells (data not shown), immunoblot analysis revealed that it did increase the amounts of total and Thr81-phosphorylated p53 protein (Figure 3A), consistent with the up-regulation of DR4 and DR5 expression (Figure 2B).

Reporter gene assays showed that ANT2 knockdown resulted in the strong induction of p53 transcriptional activity (Figure 3B). Furthermore, treatment with the p53 inhibitor pifithrin-α not only prevented the up-regulation of DR4 and DR5 by ANT2 knockdown in MCF7 cells (Figure 3C) it also blocked its TRAIL-sensitizing effect (Figure 3D). Taken together, these data suggest that ANT2 shRNA treatment stabilizes p53 and increases its transcriptional activity, thereby up-regulating DR4 and DR5 expression and priming TRAIL-resistant MCF7 cells to undergo TRAIL-induced apoptosis.

### ANT2 knockdown induces JNK activation

**Figure 4:**
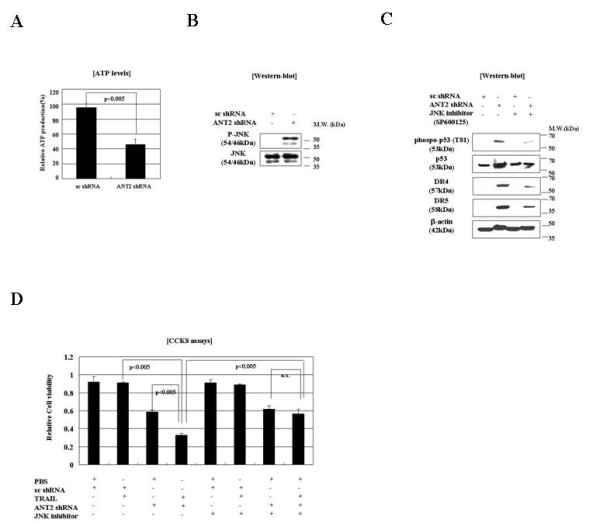
**Transfection of ANT2 shRNA leads to JNK activation and subsequent up-regulation and activation of p53 in MCF7 cells**. (A) Analysis of ATP levels in lysates of MCF7 cells pre-treated with the JNK inhibitor SP600125 for 2 h and then transfected with scrambled or ANT2 shRNA for 24 h. The data are shown in relative luminescence units (RLU) calculated by normalizing total intracellular ATP to total protein. (B) MCF7 cells treated as in (A) were analyzed for JNK phosphorylation by immunoblotting using anti-phospho JNK and anti-JNK antibodies. (C) Effect of JNK inhibition on ANT2 shRNA-mediated upregulation of DR4 and DR5. MCF7 cells were treated with SP600125 and scrambled or ANT2 shRNA as in (A). Cell extracts were subjected to SDS-PAGE and immunoblotted with antibodies against phospho-p53, p53, DR4, DR5, and β-actin (control).

As shown in Figure 3A, we observed that ANT2 shRNA transfection increased the total amount of p53 protein and the amount of Thr81-phosphorylated p53 in MCF7 cells. Because JNK is known to specifically phosphorylate p53 at Thr81 in response to stress, and because JNK is important for p53 stabilization and transcriptional activity [26], we hypothesized that JNK might be activated by cellular stress (*e.g*., ATP depletion) induced by ANT2 knockdown. As expected, ANT2 shRNA treatment depleted ATP levels by approximately half and was accompanied by JNK phosphorylation (Figure 4A and 4B). Furthermore, the phosphorylation of p53 Thr81 in ANT2 shRNA-transfected cells was inhibited by the JNK inhibitor SP600125 (Figure 4C). Taken together, these findings suggest that ANT2 knockdown activates JNK to stabilize and activate p53, which then up-regulates DR4 and DR5 expression.

### Through JNK activation, ANT2 knockdown induces DNMT1 to hypermethylate the DcR2 promoter, thereby down-regulating DcR2 expression

**Figure 5:**
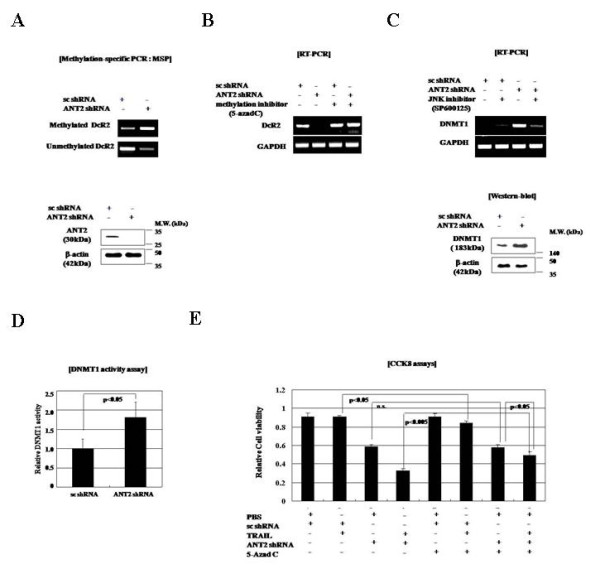
**ANT2 knockdown-induced JNK signaling up-regulates DNMT1, leading to methylation of the DcR2 promoter in MCF7 cells**. (A) Methylation-specific PCR analysis of effect of ANT2 shRNA on DcR2 genomic DNA of MCF7 cells. After cells were transfected with scrambled or ANT2 shRNA for 24 h, they were lysed, and the genomic DNA was purified. Unmethylated cytosines were converted to uracils using an Invitrogen MethylCode Bisulfite Conversion Kit, and the converted genomic DNA was used for PCR with methylation-specific primers. The cell extracts were also analyzed for ANT2 and β-actin expression by immunoblotting. (B) RT-PCR analysis of the effect of ANT2 shRNA-induced methylation on DcR2 expression. Cells were pre-treated with the methylation inhibitor 5-aza-dC for 2 h and then transfected with scrambled or ANT2 shRNA. (C) RT-PCR analysis of effect of ANT2 shRNA-induced JNK signaling on DNMT1 expression. MCF7 cells were pre-treated with SP600125 for 2 h and then transfected with scrambled or ANT2 shRNA. Cell extracts were also analyzed for DNMT1 and β-actin expression by immunoblotting. (D) Effect of ANT2 shRNA-induced JNK signaling on DNMT1 activity. MCF7 cells were treated as in (C), and nuclear extracts were evaluated for DNMT1 activity. (E) Effect of JNK inhibition on ANT2 shRNA-induced sensitization to TRAIL. MCF7 cells were treated with SP600125 and scrambled or ANT2 shRNA as in (C), and 24 h after transfection, they were treated with recombinant human TRAIL (100 ng/ml) for 12 h. Cell viability was measured using a CCK8 assay kit.

Our observation that ANT2 knockdown not only up-regulated the expression of DR4 and DR5 but also down-regulated the expression of DcR2 in MCF7 cells led us to examine the methylation status of the DcR2 promoter. In MCF7 cells, the DcR2 promoter was largely unmethylated, but in ANT2 shRNA-transfected MCF7 cells, the DcR2 promoter was hypermethylated, thereby suppressing DcR2 expression (Figure 5A). Furthermore, treatment with the demethylating agent 5-aza-dC restored DcR2 expression in ANT2-shRNA-transfected MCF7 cells (Figure 5B). These data indicate that DcR2 down-regulation in response to ANT2 knockdown is mediated by DcR2 promoter hypermethylation.

DNMT1 catalyzes the methylation of CpG islands in promoter regions, thereby contributing to gene silencing. A report that DNMT1 activation is involved in JNK signaling [27], and our observations that ANT2 knockdown activates JNK and causes hypermethylation of the DcR2 promoter in MCF7 cells, led us to investigate the role of DNMT1 in ANT2 shRNA-induced suppression of DcR2. Whiles DNMT1 protein levels were significantly increased in MCF7 cells transfected with ANT2 shRNA, this increase was blocked by the JNK inhibitor SP600125 (Figure 5C). Similarly, DNMT1 activity assays showed an SP600125-inhibitable increase in DNMT1 activity in MCF7 cells transfected with ANT2 shRNA (Figure 5D). SP600125 treatment also blocked the TRAIL-sensitizing effect of ANT2 knockdown (Figure 5E). These data suggest that ANT2 knockdown-induced JNK activation causes over-expression and activation of DNMT1 in MCF7 cells, thereby inducing hypermethylation of the DcR2 promoter and subsequent silencing of the DcR2 gene.

### ANT2 knockdown increases TRAIL sensitivity of breast cancer xenografts by regulating TRAIL receptors, thereby significantly inhibiting tumor growth *in vivo*

**Figure 6:**
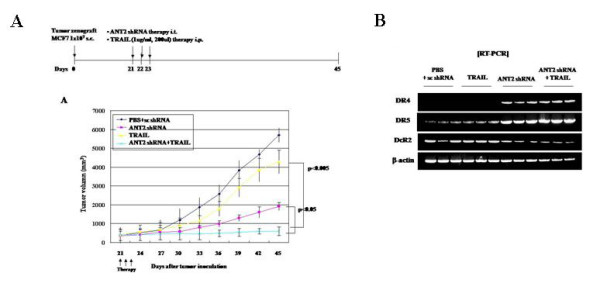
**Anti-tumor effects of ANT2 shRNA and TRAIL in a breast cancer xenograft model *in vivo***. (A) Experimental protocol for tumor challenge and shRNA and TRAIL treatment. BALB/c nude mice were challenged with 5 × 10^6 ^MCF7 cells by subcutaneous injection into the right flanks. After 21 days, they began 3 days of treatment with thrice daily intraperitoneal injections of PBS or human recombinant TRAIL with or without thrice daily intratumoral injections of Lipofectamine 2000-supplemented scrambled or ANT2 shRNA vector. Calipers were used to make weekly measurements of tumor dimensions, and tumor volumes were calculated as described in the Methods. Measurements continued until day 45 after tumor challenge. Data were analyzed using the Student *t *test. Differences are considered statistically significant at *P *< 0.05. (B) Expression of TRAIL receptors in tumors after shRNA and TRAIL treatment. Tumors were isolated on day 45 after tumor challenge and subjected to RT-PCR using primers specific for human DR4, DR5, DcR2, and GAPDH (internal control).

Because our *in vitro *results suggested that ANT2 knockdown enhances the apoptosis-inducing potential of TRAIL in MCF7 cells *in vitro*, we next examined the effects of ANT2 shRNA/TRAIL combination therapy on a breast cancer xenograft model *in vivo*. For this purpose, MCF7 cells were implanted into the right thighs of BALB/c nude mice. Three weeks after implantation, the mice began treatment with thrice daily injections of PBS or TRAIL, with or without scrambled or ANT2 shRNA, for 3 days, as described in the Methods section. The administration of TRAIL alone suppressed tumor growth by approximately 15%, whereas the administration of ANT2 shRNA alone suppressed tumor growth by approximately 65%. Of note, co-treatment with ANT2 shRNA and TRAIL had a greater effect than either treatment alone, inhibiting tumor growth by approximately 90% (Figure 6A).

Treatment of the mice with ANT2 shRNA also up-regulated DR4 and DR5 expression and down-regulated DcR2 expression in the tumor cells (Figure 6B). These findings suggest that the TRAIL-sensitizing effect of ANT2 knockdown on breast cancer cells observed *in vitro *also occurs *in vivo *via the same mechanism involving regulation of TRAIL receptors. Thus, combination therapy with ANT2 shRNA and TRAIL might be effective against breast cancer.

## Discussion

The four known human ANT isoforms (ANT1, ANT2, ANT3, and ANT4) exhibit tissue-, cell-type-, and developmental stage-specific expression patterns [18]. ANT1 is strongly expressed in terminally differentiated cells; ANT2 is overexpressed in tissues that have high proliferative ability and in several cancer cell lines [16,21]; and ANT3 is ubiquitously expressed in all tissues. Whereas ANT1 and ANT3 exert pro-apoptotic effects when overexpressed, ANT2, instead, has an anti-apoptotic function [20,28]. We previously observed that ANT2 is overexpressed in the breast cancer cell lines MCF7 and MDA-MB-231 but is absent from the non-neoplastic mammary epithelial cell line MCF10A [16]. Moreover, vector-based RNAi knockdown of ANT2 expression in MCF7 and MDA-MB-231 cells resulted in cell cycle arrest and apoptotic cell death; these events were accompanied by cellular energy (ATP) depletion, mitochondrial membrane depolarization, Bax induction, and Bcl-xL down-regulation [16]. TNF-α and TNF receptor I were also induced through a bystander cytotoxic effect. Those data suggested that suppression of ANT2 might selectively target cancer cells while having little effect on normal cells. Other researchers have also reported that ANT2 knockdown sensitizes cancer cells to a chemotherapeutic agent that targets mitochondria [21].

Apoptosis occurs via two main pathways, namely, the extrinsic death receptor and intrinsic mitochondrial pathways. Because TRAIL induces apoptosis selectively in tumor cells, TRAIL-targeting agents are considered promising candidates for cancer prevention and treatment, but their clinical application is hindered by the TRAIL-resistance of many cancer cells. Many kinds of chemotherapeutic drugs or radiotherapy have been combined with TRAIL-engaging agents to overcome TRAIL resistance via various mechanisms [29-33]. Some of these studies have presented convincing evidence that up-regulation of the DR4 and DR5 death receptors and down-regulation of the DcR1 and DcR2 decoy receptors can restore the TRAIL sensitivity of TRAIL-resistant cells [34-37]. In the present study, the resistance of MCF7 cells to TRAIL-induced apoptosis led us to investigate whether ANT2 knockdown would sensitize these cells to TRAIL. As has been previously reported [38], we found that MCF7, but not MDA-MB-231 cells, were strongly resistant to TRAIL-induced apoptosis. In line with these observations, DR4 and DR5 expression was low in MCF7 cells, and DcR2 expression was high, whereas in TRAIL-sensitive MDA-MB-231 cells, DR4 and DR5 expression were strongly expressed and DcR2 expression was very low. Of note, ANT2 knockdown by RNAi conferred TRAIL sensitivity on MCF7 cells by up-regulating DR4/DR5 and down-regulating DcR2 *in vitro *and *in vivo*. However, since ANT2 shRNA treatment also affects the expression of Bax and Bcl-xL [16], the ANT2 shRNA-induced sensitization of MCF7 cells to TRAIL might involve alterations in the expression of other molecules, such as FLIP, Bcl2-family proteins, and XIAPs, that regulate TRAIL-mediated apoptosis.

JNK is activated by many cellular stresses, including ultraviolet irradiation, oxidative stresses, inflammatory cytokines, growth factor withdrawal, and DNA-damaging agents (*e.g.*, cisplatin, etoposide, and doxorubicin) [39]. JNK plays important roles in cell proliferation/survival and apoptosis, and whether JNK activation results in cell proliferation or apoptosis depends on the stimulus, the cell type, the duration and strength of the activation signal, and other factors [40]. JNK up-regulates pro-apoptotic genes and down-regulates anti-apoptotic genes through the transactivation of several transcription factors, including c-Jun and p53 [40]. Our study is the first to characterize the effect of ANT2 on death receptor-mediated apoptosis. We found that ANT2 knockdown activates JNK and that this activation is indispensable for ANT2 shRNA-induced sensitization of MCF7 cells to TRAIL. The activation of JNK led to phosphorylation of p53 at Thr81, the consequent stabilization and transcriptional activation of p53, and the previously well-characterized up-regulation of DR4 and DR5 expression by activated p53.

Activation of JNK also induced DNMT1 expression in ANT2 shRNA-treated MCF7 cells, although DNMT1 expression was not detected in untreated MCF7 cells. This increased DNMT1 activity led to hypermethylation of the DcR2 promoter, thereby suppressing DcR2 expression. Treatment of ANT2-knockdown MCF7 cells with a JNK inhibitor prevented DNMT1 induction. The loss of cellular energy (ATP) caused by ANT2 knockdown and the resulting oxidative stress [41] might trigger JNK activation in order to stimulate apoptotic signaling.

Our experiments using a tumor xenograft model showed that ANT2-knockdown-induced sensitization to TRAIL also occurs *in vivo *and that combination therapy with ANT2 shRNA and TRAIL very efficiently inhibits tumor growth. Our findings that the novel anti-cancer effects of ANT2 knockdown in breast cancer cells involves TRAIL sensitization through the regulation of TRAIL receptors via JNK activation and subsequent increase of p53 activity and DNMT1 expression suggest that ANT2 suppression by shRNA might be useful as a new strategy for overcoming TRAIL-resistance in cancer therapy.

## Conclusions

Our findings suggest a new therapeutic strategy for breast cancer. The use of ANT2 shRNA in combination with TRAIL up-regulates DR4/DR5 expression and down-regulates DcR2 expression *in vitro *and *in vivo*, inducing apoptosis in a TRAIL-resistant human breast cancer cell line and enhancing survival in a tumor-bearing mouse model. Even by itself, ANT2 shRNA has an anticancer effect in breast cancer cell lines, acting through a mechanism involving the induction of p53 activity, JNK activation, DNMT1 expression, DR4/DR5 up-regulation, and DcR2 down-regulation, thereby making the cells susceptible to TRAIL-induced apoptosis. This study provides a foundation for the development of combinatorial treatment regimens that enhance tumor cell apoptosis. Furthermore, it shows that ANT2 shRNA acts as a chemosensitizer when administered with TRAIL, and that this chemosensitizing effect can overcome TRAIL resistance in p53-wild-type cancers. However, DR4/DR5 up-regulation also occurs after ANT2 knockdown in the p53-mutant cell lines BT474 and T47 D, demonstrating that p53 is not a necessary component of the ANT2-knockdown effect in these cells.

## Abbreviations

ANT2: adenine nucleotide translocator-2; COX: cyclooxygenase; DcR2: TRAIL decoy receptor 2; DNMT1: DNA methyl transferase I; DR4: TRAIL death receptor 4; DR5: TRAIL death receptor 5; EDTA: ethylenediamine tetra acetic acid; FITC: fluorescence isothiocyanate; FLIP: FLICE-like inhibitory protein (FLIP); GAPDH: glyceraldehyde phosphate dehydrogenase; JNK: c-Jun N-terminal kinase; PAGE: polyacrylamide gel electrophoresis; PARP: poly(ADP-ribose) polymerase (PARP); PBS: phosphate-buffered saline; PCR: polymerase chain reaction; PI, propidium iodide; RNAi: RNA interference; RT: reverse transcription; SDS: sodium dodecyl sulfate; shRNA: short-hairpin RNA; siRNA: small-interfering RNA; TNF: tumor necrosis factor; TRAIL: tumor necrosis factor-related apoptosis-inducing ligand; XIAP: X-linked inhibitor of apoptosis proteins; 5-aza-dC: 5-aza-2'-deoxycytidine.

## Competing interests

The authors have applied for a domestic patent and will apply for an international patent regarding the utilization of ANT2 siRNA technology as a therapeutic method for cancer. Seoul National University College of Medicine will retain the patent. The authors declare that they have no other competing interests.

## Authors' contributions

JYJ performed most of the experiments and worked together with YKJ to analyze the data and prepare the manuscript. YC conducted additional experiments. CWK contributed to the design of the project, to data analysis, and to writing of the paper. All authors reviewed and approved the final manuscript.

## References

[B1] Hall MA Cleveland JL Clearing the TRAIL for cancer therapy Cancer Cell 2007 12 4 6 10.1016/j.ccr.2007.06.011 17613431

[B2] Wang S El-Deiry WS TRAIL and apoptosis induction by TNF-family death receptors Oncogene 2003 22 8628 8633 10.1038/sj.onc.1207232 14634624

[B3] Huang Y Sheikh MS TRAIL death receptors and cancer therapeutics Toxicol Appl Pharmacol 2007 224 284 289 10.1016/j.taap.2006.12.007 17240413

[B4] Duiker EW Mom CH de Jong S Willemse PH Gietema JA van der Zee AG de Vries EGE The clinical trail of TRAIL Eur J Cancer 2006 42 2233 2240 10.1016/j.ejca.2006.03.018 16884904

[B5] Dyer MJ MacFarlane M Cohen GM Barriers to effective TRAIL-targeted therapy of malignancy J Clin Oncol 2007 25 4505 4506 10.1200/JCO.2007.13.1011 17906217

[B6] LeBlanc HN Ashkenazi A Apo2L/TRAIL and its death and decoy receptors Cell Death Differ 2003 10 66 75 10.1038/sj.cdd.4401187 12655296

[B7] Mahalingam D Szegezdi E Keane M de Jong S Samali A TRAIL receptor signalling and modulation: Are we on the right TRAIL? Cancer Treat Rev 2009 35 280 288 10.1016/j.ctrv.2008.11.006 19117685

[B8] Inoue S MacFarlane M Harper N Wheat LM Dyer MJ Cohen GM Histone deacetylase inhibitors potentiate TNF-related apoptosis-inducing ligand (TRAIL)-induced apoptosis in lymphoid malignancies Cell Death Differ 2004 11 Suppl 2 S193 S206 10.1038/sj.cdd.4401535 15608694

[B9] Palacios C Yerbes R López-Rivas A Flavopiridol induces cellular FLICE-inhibitory protein degradation by the proteasome and promotes TRAIL-induced early signaling and apoptosis in breast tumor cells Cancer Res 2006 66 8858 8869 10.1158/0008-5472.CAN-06-0808 16951203

[B10] Ganten TM Koschny R Haas TL Sykora J Li-Weber M Herzer K Walczak H Proteasome inhibition sensitizes hepatocellular carcinoma cells, but not human hepatocytes, to TRAIL Hepatology 2005 42 588 597 10.1002/hep.20807 16037944

[B11] Ricci S Kim SH Ogi K Plastaras JP Ling J Wang W Jin Z Liu YY Dicker DT Chiao PJ Flaherty KT Smith CD El-Deiry WS Reduction of TRAIL-induced Mcl-1 and cIAP2 by c-Myc or sorafenib sensitizes resistant human cancer cells to TRAIL-induced death Cancer Cell 2007 12 66 80 10.1016/j.ccr.2007.05.006 17613437

[B12] Shi RX Ong CN Shen HM Protein kinase C inhibition and X-linked inhibitor of apoptosis protein degradation contribute to the sensitization effect of luteolin on tumor necrosis factor-related apoptosis-inducing ligand-induced apoptosis in cancer cells Cancer Res 2005 65 7815 23 10.1158/0008-5472.CAN-04-2749 16140950

[B13] Zhang S Shen HM Ong CN Down-regulation of c-FLIP contributes to the sensitization effect of 3,3-diindolylmethane on TRAIL-induced apoptosis in cancer cells Mol Cancer Ther 2005 4 1972 81 10.1158/1535-7163.MCT-05-0249 16373712

[B14] Gross A McDonnell JM Korsmeyer SJ BCL-2 family members and the mitochondria in apoptosis Genes Dev 1999 13 1899 1911 10.1101/gad.13.15.1899 10444588

[B15] Suliman A Lam A Datta R Srivastava RK Intracellular mechanisms of TRAIL: apoptosis through mitochondrial-dependent and -independent pathways Oncogene 2001 20 2122 33 10.1038/sj.onc.1204282 11360196

[B16] Jang JY Jeon YK Kim CW Suppression of adenine nucleotide translocase-2 by vector-based siRNA in human breast cancer cells induces apoptosis and inhibits tumor growth *in vitro *and *in vivo* Breast Cancer Research 2008 10 R11 10.1186/bcr1857 18267033 PMC2374967

[B17] Jänicke RU MCF-7 breast carcinoma cells do not express caspase-3 Breast Cancer Res Treat 2009 117 219 221 10.1007/s10549-008-0217-9 18853248

[B18] Doerner A Pauschinger M Badorff A Noutsias M Giessn S Schulze K Bilger J Rauch U Schultheiss HP Tissue-specific transcription pattern of the adenine nucleotide translocase isoforms in humans FEBS Lett 1997 414 258 62 10.1016/S0014-5793(97)01000-4 9315697

[B19] Luciakova K Barath P Poliakova D Persson A Nelson BD Repression of the human adenine nucleotide translocase-2 gene in growth-arrested human diploid cells J Biol Chem 2003 278 30624 33 10.1074/jbc.M303530200 12777383

[B20] Stepien G Torroni A Chung AB Hodge JA Wallace DC Differential expression of adenine nucleotide translocator isoforms in mammalian tissues and during muscle cell differentiation J Biol Chem 1992 267 14592 97 1378836

[B21] Le Bras M Borgne-Sanchez A Touat Z El Dein OS Deniaud A Maillier E Lecellier G Rebouillat D Lemair C Kroemer G Jacotot E Brenner C Chemosensitization by knockdown of adenine nucleotide translocase-2 Cancer Res 2006 66 9143 52 10.1158/0008-5472.CAN-05-4407 16982757

[B22] Faure Vigny H Heddi A Giraud S Chautard D Stepien G Expression of oxidative phosphorylation genes in renal tumors and tumoral cell lines Mol Carcinog 1996 16 165 72 10.1002/(SICI)1098-2744(199607)16:3<165::AID-MC7>3.0.CO;2-G 8688152

[B23] Chevrollier A Loiseau D Chabi B Renier G Donay O Malthiery Y Stepien G ANT2 isoform required for cancer cell glycolysis J Bioenerg Biomembr 2005 37 307 16 10.1007/s10863-005-8642-5 16341775

[B24] Chevrollier A Loiseau D Gautier F Malthlery Y Stepien G ANT2 expression under hypoxic conditions produces opposite cell cycle behavior in 143B and HepG2 cancer cells Mol Carcinog 2005 42 1 8 10.1002/mc.20059 15486956

[B25] Chevrollier A Loiseau D Stepien G What is the specific role of ANT2 in cancer cells? Med Sci (Paris) 2005 21 156 61 15691486 10.1051/medsci/2005212156

[B26] Wu GS Burns TF McDonald ER Jiang W Meng R Krantz ID Kao G Gan DD Zhou JY Muschel R Hamilton SR Spinner NB Markowitz S Wu G el-Deiry WS KILLER/DR5 is a DNA damage-inducible p53-regulated death receptor gene Nat Genet 1997 17 2 141 3 10.1038/ng1097-141 9326928

[B27] Buschmann T Potapova O Bar-Shira A Ivanov VN Fuchs SY Henderson S Fried VA Minamoto T Alarcon-Vargas D Pincus MR Gaarde WA Holbrook NJ Shiloh Y Ronai Z Jun NH_2_-terminal kinase phosphorylation of p53 on Thr-81 is important for p53 stabilization and transcriptional activities in response to stress Mol Cell Biol 2001 21 8 2743 54 10.1128/MCB.21.8.2743-2754.2001 11283254 PMC86905

[B28] Bauer MK Schubert A Rocks O Grimm S Adenine nucleotide translocase-1, a component of the permeability transition pore, can dominantly induce apoptosis J Cell Biol 1999 147 1493 1502 10.1083/jcb.147.7.1493 10613907 PMC2174250

[B29] Shankar S Chen X Srivastava RK Effects of sequential treatments with chemotherapeutic drugs followed by TRAIL on prostate cancer *in vitro *and *in vivo* Prostate 2005 62 165 86 10.1002/pros.20126 15389801

[B30] Shankar S Singh TR Chen X Thakkar H Firnin J Srivastava RK The sequential treatment with ionizing radiation followed by TRAIL/Apo-2L reduces tumor growth and induces apoptosis of breast tumor xenografts in nude mice Int J Oncol 2004 24 1133 40 15067334

[B31] Shankar S Singh TR Fandy TE Luetrakul T Ross DD Srivastava RK Interactive effects of histone deacetylase inhibitors and TRAIL on apoptosis in human leukemia cells: involvement of both death receptor and mitochondrial pathways Int J Mol Med 2005 16 1125 38 16273296

[B32] Shankar S Singh TR Srivastava RK Ionizing radiation enhances the therapeutic potential of TRAIL in prostate cancer *in vitro *and *in vivo*: intracellular mechanisms Prostate 2004 61 35 49 10.1002/pros.20069 15287092

[B33] Shankar S Srivastava RK Enhancement of therapeutic potential of TRAIL by cancer chemotherapy and irradiation: mechanisms and clinical implications Drug Resist Updat 2004 7 139 56 10.1016/j.drup.2004.03.002 15158769

[B34] Galligan L Longley DB McEwan M Wilson TR McLaughlin K Johnston PG Chemotherapy and TRAIL-mediated colon cancer cell death: the roles of p53, TRAIL receptors, and c-FLIP Mol Cancer Ther 2005 4 2026 36 10.1158/1535-7163.MCT-05-0262 16373718

[B35] Jin Z McDonald ER III Dicker DT El-Deiry WS Deficient tumor necrosis factor-related apoptosis-inducing ligand (TRAIL) death receptor transport to the cell surface in human colon cancer cells selected for resistance to TRAIL-induced apoptosis J Biol Chem 2004 279 35829 39 10.1074/jbc.M405538200 15155747

[B36] Meng RD McDonald ER Sheikh MS Fornace AJJ El-Deiry WS The TRAIL decoy receptor TRUNDD (DcR2, TRAIL-R4) is induced by adenovirus-p53 overexpression and can delay TRAIL-, p53-, and KILLER/DR5-dependent colon cancer apoptosis Mol Ther 2000 1 130 44 10.1006/mthe.2000.0025 10933923

[B37] Pan G Ni J Wei YF Yu G Gentz R Dixit VM An antagonist decoy receptor and a death domain-containing receptor for TRAIL Science 1997 277 815 18 10.1126/science.277.5327.815 9242610

[B38] Sanlioglu A Dirice E Aydin C Erin N Koksoy S Sanlioglu S Surface TRAIL decoy receptor-4 expression is correlated with TRAIL resistance in MCF7 breast cancer cells BMC Cancer 2005 5 54 72 10.1186/1471-2407-5-54 15916713 PMC1156874

[B39] Ouyang DY Wang YY Zheng YT Activation of c-Jun N-terminal kinases by ribotoxic stresses Cell Mol Immunol 2005 2 419 425 16426491

[B40] Dhanasekaran DN Reddy EP JNK signaling in apoptosis Oncogene 2008 27 6245 6251 10.1038/onc.2008.301 18931691 PMC3063296

[B41] Yaglom JA Ekhterae D Gabai VL Sherman MY Regulation of necrosis of H9c2 myogenic cells upon transient energy deprivation: rapid deenergization of mitochondria precedes necrosis and is controlled by reactive oxygen species, stress kinase JNK, HSP72 and ARC J Biol Chem 2003 278 50483 50496 10.1074/jbc.M306903200 14523009

